# The current practice of handling and reporting missing outcome data in eight widely used PROMs in RCT publications: a review of the current literature

**DOI:** 10.1007/s11136-015-1206-1

**Published:** 2016-01-28

**Authors:** Ines Rombach, Oliver Rivero-Arias, Alastair M. Gray, Crispin Jenkinson, Órlaith Burke

**Affiliations:** Health Economics Research Centre (HERC), Nuffield Department of Population Health, University of Oxford, Oxford, UK; RCS Surgical Intervention Trials Unit (SITU), Nuffield Department of Orthopaedics, Rheumatology and Musculoskeletal Sciences, University of Oxford, Oxford, UK; National Perinatal Epidemiology Unit (NPEU), Nuffield Department of Population Health, University of Oxford, Oxford, UK; Health Services Research Unit (HSRU), Nuffield Department of Population Health, University of Oxford, Oxford, UK; Clinical Trial Service Unit and Epidemiological Studies Unit (CTSU), Nuffield Department of Population Health, University of Oxford, Oxford, UK

**Keywords:** Missing data, Randomised controlled trials (RCTs), Patient-reported outcomes measures (PROMs), Quality of life (QoL), Sensitivity analysis

## Abstract

**Purpose:**

Patient-reported outcome measures (PROMs) are designed to assess patients’ perceived health states or health-related quality of life. However, PROMs are susceptible to missing data, which can affect the validity of conclusions from randomised controlled trials (RCTs). This review aims to assess current practice in the handling, analysis and reporting of missing PROMs outcome data in RCTs compared to contemporary methodology and guidance.

**Methods:**

This structured review of the literature includes RCTs with a minimum of 50 participants per arm. Studies using the EQ-5D-3L, EORTC QLQ-C30, SF-12 and SF-36 were included if published in 2013; those using the less commonly implemented HUI, OHS, OKS and PDQ were included if published between 2009 and 2013.

**Results:**

The review included 237 records (4–76 per relevant PROM). Complete case analysis and single imputation were commonly used in 33 and 15 % of publications, respectively. Multiple imputation was reported for 9 % of the PROMs reviewed. The majority of publications (93 %) failed to describe the assumed missing data mechanism, while low numbers of papers reported methods to minimise missing data (23 %), performed sensitivity analyses (22 %) or discussed the potential influence of missing data on results (16 %).

**Conclusions:**

Considerable discrepancy exists between approved methodology and current practice in handling, analysis and reporting of missing PROMs outcome data in RCTs. Greater awareness is needed for the potential biases introduced by inappropriate handling of missing data, as well as the importance of sensitivity analysis and clear reporting to enable appropriate assessments of treatment effects and conclusions from RCTs.

**Electronic supplementary material:**

The online version of this article (doi:10.1007/s11136-015-1206-1) contains supplementary material, which is available to authorized users.

## Background

Over the last 20 years, clinicians and policy makers have increasingly become aware of the importance of incorporating the patient perspective to inform patient care and policy decisions [1, 2]. As a consequence, a large number of instruments have been developed to collect information on patients’ perceived health states or their perceived health-related quality of life (HRQOL) [3, 4]. Often referred to as patient-reported outcomes (PROs) or patient-reported outcome measures (PROMs), these measures include ‘any report coming directly from patients, without interpretation by physicians or others, about how they (the patients) function or feel in relation to a health condition and its therapy’ [5].

PROMs are an important addition to traditional measures of outcome, such as clinical assessment, morbidity and mortality, which may not fully capture the patient experience of a specific treatment or disease burden. Therefore, PROs are increasingly used as primary and secondary endpoints in randomised controlled trials (RCTs) [1, 2].

However, RCTs utilising PROMs rely on their participants to be able and willing to complete the relevant outcome measures throughout their follow-up period. It is therefore often impossible to obtain complete follow-up PROMs data for all randomised participants [6], and the subsequently arising missing data within those RCTs can question their ability to provide reliable patient-reported effectiveness and cost-effectiveness estimates of potential interventions [7].

### Missing data background

Missing data are defined as data that were intended to be collected within the remit of a study, and considered relevant to the statistical analysis and interpretation of the results, but which are unavailable at the time of the analysis [8].

Statistical methodology commonly refers to three missing data mechanisms, which were first defined by Little and Rubin in 1987 [9]. In simple terms, they describe if the probability of an observation being missing is (1) unrelated to any of the observed or unobserved data (missing completely at random—MCAR), (2) related to the observed data (missing at random—MAR) and (3) related to the unobserved outcome data (missing not at random—MNAR).

Based on the available data, it is impossible to definitively assign one of these missing data mechanisms to the data. Yet, if the assumed mechanism is not correct, the results from the statistical analysis may be biased [10], making it imperative to perform adequate sensitivity analyses which vary the assumptions made in the primary analysis about the underlying missing data mechanism [11].

### Overview of statistical approaches to missing data

Various approaches have been developed for handling missing data in statistical analyses, which can be divided into the following categories [12, 13]: (1) available/complete case analysis excludes all observations with missing data in any of the relevant variables; (2) single imputation techniques replace the missing value with a value based on either previously observed data for that individual (last observation carried forward—LOCF), the mean of available data (mean imputation) or informed by a range of other variables (regression imputation); (3) multiple imputation techniques are drawn on other observed data to impute a range of possible values; separate analysis models are run for each of these imputed values and pooled to take into account the uncertainty around the missing data; and (4) model-based approaches include maximum likelihood methods and mixed-effects models for longitudinal data, which do not require the imputation of missing values.

Whether RCT results are biased due to the occurrence of missing data, and how much bias is introduced as a result depends on a multitude of factors, mainly the extent of missing data within the study and within each trial arm, the appropriateness of the assumptions made about the underlying missing data mechanism and the subsequent handling of the missing data in the analysis [6]. Analyses will be unbiased under MCAR, and also under MAR if the analysis adjusts for all variables the probability of missing data is related to, although the power of the study is decreased due to the reduced sample size.

RCTs form the basis for many important healthcare decisions [7], such as the approval of new or modified drugs, devices or interventions, and changes to clinical guidelines or practice [14]. If these decisions are informed by biased data, due to the inappropriate handling and reporting of missing data within the underlying RCTs, this could lead to substandard or even harmful treatments being recommended and adversely affect patient welfare.

Previous reviews [15–22] have identified substandard handling and reporting of missing primary outcome data in RCTs and epidemiological studies, the use of inappropriate methods to account for missing data and the lack of sensitivity analyses to assess the robustness of study results, all highlighting the need for clearer reporting of missing data within studies.

The literature on how missing data should be handled and reported is manifold and covers methods of imputation [11, 23–26], analysis methods [9, 11, 12] and reporting standards [14, 27–29]. However, specific advice on handling missing PROMs data is less common. A systematic review and Delphi consensus by Li et al. [10] consolidated the literature into a set of ten standards that should be applied for the prevention and handling of missing data in research utilising PROMs.

### Aims of this review

This work aims to:

- Create an overview of the current practice of handling, analysis and reporting of missing PROMs outcome data (including both primary and secondary endpoints) in journal publications of RCTs, thus updating previous reviews.
- Compare the currently used methods to handle, analyse and report missing PROMs outcome data in RCTs against recommended best practice.

## Methods

### Basis for the comparison

Assessment of study design, analysis and reporting in the review was based on seven of the ten criteria recommended by Li et al. [10], as listed in Table 1. The remaining three criteria related to study design (clear definition of research question and primary endpoints) and study conduct (continued collection of key outcomes and monitoring of missing data) were outside the remit of this review as they relate to the protocol and internal trial conduct and may therefore not be directly assessable based on the publications reporting on trial results.

**Table 1:** Reporting standards defined by Li et al. assessed in this review

Proposed standards	Aspects assessed within the literature review
Standards on study design	Steps have been taken and reported to conduct the study in a way to minimise missing data
Standards on analysis	Single imputation methods are avoided The analytical and/or imputation methods used are able to account for the uncertainty associated with missing data Appropriate sensitivity analysis examines the robustness of results with regard to the assumptions about the missing data mechanism
Standards on reporting	All randomised participants are accounted for in the results Appropriate reporting of the extent of missing data and methods to handle it Discussion of the potential influence of missing data on the study results

When designing this review, it was felt important to include questionnaires from four key PROMs areas, namely preference-based measures (which can be used in health economics evaluations), generic health profiles, disease-targeted questionnaires and anatomical site-specific questionnaires. Two PROMs within each category were selected, using the criteria that they were validated and had been widely adopted and that they aligned with the authors’ research interests and experience:

- Utility measures: EuroQol EQ-5D-3L Questionnaire [30, 31] and Health Utility Index (HUI) [32], whereby articles utilising any of the available HUI versions (including HUI-1, HUI-2 and HUI-3) were eligible for inclusion.
- Generic health profiles: Short-Form 12 (SF-12) [33] and Short-Form 36 (SF-36) [34] health surveys.
- Site-specific questionnaires: Oxford Hip Score (OHS) [35, 36] and Oxford Knee Score (OKS) [36, 37].
- Disease-targeted questionnaires: European Organization for Research and Treatment of Cancer Quality of Life Questionnaire-Core 30 (EORTC QLQ-C30) [38] and Parkinson Disease Questionnaire (a combination of the PDQ-8 and PDQ-39 was considered) [39, 40].

### Database search

**Fig. 1:**
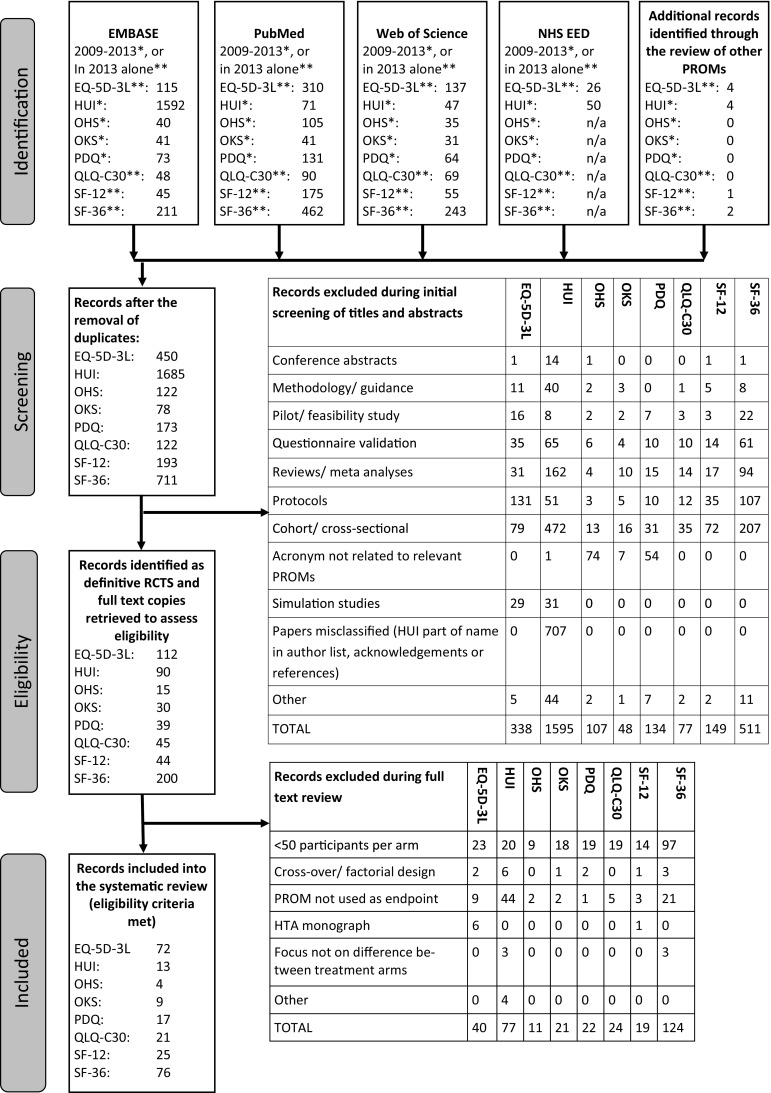
PRISMA flow diagramm detailing the identification process of studies for inclusion in the review

Multiple databases [EMBASE, PubMed, Web of Science, NHS Economic Evaluation Database (NHS EED, for the two preference-based measures only)] were searched to identify recent publications of RCT results utilising at least one relevant questionnaire as either a primary or secondary endpoint. To minimise the risk of missing potentially relevant articles, very general search terms were used to identify publications, using the words (random*) and (clinical* or trial or RCT) and terms describing the relevant questionnaire names or abbreviations. Figure 1 depicts the number of articles identified in the initial searches, the screening process and the identification of eligible papers.

#### Eligibility of articles

Publications were considered eligible if the results from definitive RCTs utilising relevant PROMs were reported in English and at least 50 patients were randomised to each of the relevant trial arms. This cut-off was chosen to include studies of sufficient size to have permitted the use of potentially complex methods of handling missing data and quantitative assessments between treatment arms; the generalisability from smaller studies is likely to be unreliable. Due to large numbers of articles identified, searches were restricted to 2013 for the EQ-5D-3L, QLQ-C30 and SF-12, SF-36, while data extraction was extended to include years 2009–2013 for the HUI, OHS, OKS and PDQ.

Publications reporting cost-effectiveness analyses alongside clinical trials and using EQ-5D-3L or HUI data were included, but publications based primarily on extrapolations beyond the trial follow-up or on decision analytical models were excluded, as were publications reporting on aggregate data from two or more studies. Crossover studies were excluded from this review as the impact on each missing observation is greater compared to a parallel group design, and RCTs analysed within a factorial design framework were excluded as the analytical methods employed tend to differ from those for parallel group designs and may make the imputation of missing values more challenging. Most of the identified trials allocated participants to two groups. Trials with more than two arms were included in the review; however, for summaries relying on the direct comparison between two arms as well as the sample size, only two arms of the multi-arm trials were considered (i.e. the arm using the combination of most drugs or most frequent intervention appointments and the control arm).

#### Data extraction

Information was extracted from each eligible research article on study characteristics and adherence with reporting items recommended by Li et al. [10]. A full list of items extracted can be seen in the electronic supplementary material.

Data extraction was performed by one investigator (IR), with queries resolved by consultation with the other authors. Abstracts and methods sections were read in full, while a keyword search was used to identify relevant information in other sections of the articles.

Findings were summarised descriptively overall and by PROM using frequency and percentages for categorical data and medians, interquartile range and range for continuous data.

## Results

The number of identified eligible studies varied widely, from over 70 studies using the EQ-5D-3L index and SF-36 identified in 2013 alone, to less than ten studies utilising the OKS and OHS identified between 2009 and 2013, as shown in Fig. 1. Where an eligible publication reports on several of the pre-specified outcome measures, this study is included in the summaries for all relevant PROMs and more than once in the overall summaries (i.e. this review includes 237 records relating to 209 articles).

Table 2 shows that the sample size of the RCTs included into this review also varies, from a total sample size of 100 (the cut-off for eligibility to be included into the review, i.e. at least 50 participants in both of the two relevant trial arms), up to over 18,000 participants randomised across 43 countries.

**Table 2:** Overview of the characteristics of the identified RCTs by PROM category

Questionnaires	EQ-5D-3L index	HUI	OHS	OKS	PDQ	QLQ-C30	SF-12	SF-36	Overall
Number of studies	72	13	4	9	17	21	25	76	237
Years included	2013	2009–2013	2009–2013	2009–2013	2009–2013	2013	2013	2013	2009–2013
Studies using PROM as a primary outcome (%)	38.9	69.2	25.0	44.4	41.2	23.8	24.0	26.3	33.8
Size of studies^a^									
Median	329	255	155	165	294	309	241	202	251
IQR	190–600	139,622	n/a	120–200	184–359	178–420	195–392	138–304	159–416
Range	100–18,624	104–762	126–161	100–1715	109–586	108–1528	116–1573	100–3.057	100–18,624
Follow-up data is measured repeatedly (opposed to once only) (%)	87.5	92.3	50.0	88.9	76.47	81.0	84.0	77.6	82.3
Length of follow-up to primary assessment time point (in months)									
Median	12	12	18	12	6	12	9	15	12
IQR	6–17	10.5–22	7.5–36	12–24	4–10.5	6–12	6–15	4–12	6–15
Range	1–60	6–36	3–48	3–60	3–36	0.25–78	1.5–24	0.75–60	0.25–78

^a^The size of the studies described here refers to the number of participants randomised to the two relevant treatment arms considered in this review

The percentage of studies using the relevant PROMs as a primary outcome measure was highest for those utilising the HUI with almost 70 % and lowest for the OHS, QLQ-C30, SF-12 and SF-36 with approximately 25 %. RCTs using the QLQ-C30 often favoured primary endpoints focussing on survival or progression-free survival, while RCTs utilising the SF-36 often used primary endpoints that were more disease targeted. Alternative site-specific instruments may have been used as primary endpoints in RCTs that utilised the OHS or OKS. Outcomes were measured repeatedly during the follow-up period in the vast majority of studies (82 % on average). Studies with a single follow-up time point often had a very short duration of follow-up.

Full details of the study characteristics are given in Table 2.

### Missing data within the identified publications

On average, only 40 % of studies clearly stated the number of participants for whom relevant PROMs data were available at the main follow-up point; overall, approximately 37 % of all studies reported this information by randomisation allocation.

The median percentage of available PROMs data at the primary assessment time point, where reported, was 75 %, although data availability ranged from <30 to 99 %. Evidence of differential loss of follow-up between the trial arms was observed, with up to 15 % more data being missing in either trial arm, as reported in Table 3.

**Table 3:** Overview of the amount of missing data within the identified RCTs by PROM category

Questionnaires	EQ-5D-3L index	HUI	OHS	OKS	PDQ	QLQ-C30	SF-12	SF-36	Overall
Number of studies	72	13	4	9	17	21	25	76	237
% of data available at primary analysis time point (overall)^a^	(*n* = 37, 51.4 %)	(*n* = 3, 23.1 %)	(*n* = 2, 50.0 %)	(*n* = 4, 44.4 %)	(*n* = 4, 23.5 %)	(*n* = 10, 47.6 %)	(*n* = 10, 40.0 %)	(*n* = 24, 31.6 %)	(*n* = 95, 40.1 %)
Median (%)	74.8	76.2	63.3	83.7	83.2	50.7	68.6	84.2	75.0
IQR (%)	59.7–85.7					47.6–74.6	61.9–80.8	69.7–94.7	57.1–86.2
Range (%)	34.1–91.6	50.7–86.2	55.9–70.7	62.4–98.8	51.8–94.5	35.1–85.4	37.1–90.5	26.0–99.2	26.0–99.2
% difference in follow-up data (%) available (active control)^a^	(*n* = 35, 48.6 %)	(*n* = 3, 23.1 %)	(*n* = 2, 50.0 %)	(*n* = 4, 44.4 %)	(*n* = 3, 17.6 %)	(*n* = 7, 33.3 %)	(*n* = 9, 36.0 %)	(*n* = 24, 31.6 %)	(*n* = 87, 36.7 %)
Median	0.3	3.7	−2.0	−2.2	4.91	6.6	5.1	−0.5	0.3
IQR (%)	−4.0 to 4.0					2.4 to 12.3	−5.2 to 7.7	−3.6 to 2.0	−3.2 to 5.1
Range (%)	−15.7 to 10.9	−1.8 to 6.37		−3.0 to 9.4	−3.2 to 9.6	−13.1 to 13.9	−12.9 to 11.5	−13.4 to 13.9	−15.7 to 13.9

^a^The first lines of the summaries specify the number (and percentage) of studies for which this information is available

### Reporting and handling of missing data within the identified publications

Full details on the approaches to handling missing data are given in Table 4. With the exception of RCTs using the OHS and SF-12, only one-quarter or less of publications mentioned the use of strategies employed to minimise the occurrence of missing data within the study. Reported strategies to increase response rates included the provision of prepaid envelopes to increase returns of postal questionnaires, alternative assessments where clinic visit could not be attended (e.g. postal questionnaires, telephone interviews, home visits), as well as reminders where follow-up data were not received (i.e. emails, phone calls, letters). Other approaches involved payments or rewards for questionnaire completion, reiterations to participants and staff that data collection was encouraged even after withdrawal from the allocated intervention and the exclusion of potential participants that were unlikely or unable to comply with follow-up visits, including those with terminal diagnosis or hospice care.

**Table 4:** Overview of the approaches to handling missing data within the identified RCTs by PROM category

Questionnaires	EQ-5D-3L index	HUI	OHS	OKS	PDQ	QLQ-C30	SF-12	SF-36	Overall
Number of studies	72	13	4	9	17	21	25	76	237
Methods to limit missing data described (%)	25.0	15.4	50.0	22.2	11.8	14.3	36.0	21.1	22.8
Differential missingness assessed (%)^a^	25.0	15.4	0	11.1	11.8	14.3	28.0	18.4	19.8
Assumed missing data mechanism									
Not described (%)	91.7	100	100	100	82.4	100	88.0	96.0	93.7
Missing at random (%)	6.9	–	–	–	17.6	–	12.0	4.0	6.3
Missing completely at random (%)	1.4	–	–	–	–	–	–	–	0.42
Missing data mentioned in methods/analysis section (%)	62.5	53.9	25.0	11.1	75.0	42.9	52.0	52.6	54.2
Analysis population									
Intention to treat (%)	27.8	7.7	–	11.1	29.4	9.5	24.0	19.7	21.1
Modified intention to treat (%)	54.2	46.2	50.0	66.7	47.1	59.1	48.0	46.1	50.6
Per protocol (%)	1.4	–	–	–	5.9	–	–	1.3	1.3
Unclear (%)	16.7	46.2	50.0	22.2	17.7	33.3	28.0	32.9	27.0
Primary method of handling with missing data									
Complete cases (%)	38.9	30.8	50.0	22.2	5.9	14.3	32.0	39.5	32.9
Last observation carried forward (%)	11.1	7.7	–	11.1	41.2	9.5	4.0	10.5	11.8
Mean imputation (%)	5.6	–	–	–	–	–	4.0	2.7	3.0
Regression imputation (%)	–	–	–	–	–	–	4.0	–	0.4
Direct likelihood analysis (%)	–	–	–	–	5.9	–	–	–	0.4
Repeated measures model (%)	8.3	15.4	–	11.1	17.7	14.3	20.0	25.0	16.9
Multiple imputation (%)	15.3	15.4	–	–	–	–	16.0	5.3	8.9
Unclear (%)	20.8	30.8	50.0	55.6	29.4	61.9	20.0	17.1	26.2
Justification provided for primary method of dealing with missing data (%)	13.9	15.4	25.0	0	11.8	0	8.0	5.3	8.9
Sensitivity analysis was performed (%)	25.0	23.1	25.0	0	17.7	19.1	32.0	19.7	21.9
Potential influence of missing data on results mentioned in discussion (%)	18.1	15.4	25.0	0	17.7	14.3	16.0	14.5	15.6

^a^The studies considered differences between those with complete and missing data in terms of participant (baseline) characteristics

The vast majority of publications (more than 90 % overall) did not state the assumed missing data mechanism, and the relationship of missing data to baseline characteristics was rarely investigated (20 % of publications overall). In many cases, the analysis population was not clearly described (27 % of publications overall).

Many authors (17–62 %) did not clearly describe the primary method of handling missing data in the analysis. Complete case analysis was the most widely used analytic approach found in this set of publications (6–50 %). Multiple imputation and repeated measures models were less frequently used, in up to 16 and 25 % of publications, respectively.

A small number of authors justified their primary method of dealing with missing data (between 0 and 25 % across the PROMs examined), reported sensitivity analysis to assess the robustness of their results with regard to the assumed missing data mechanism (0–32 %) or commented on the potential influence of missing data on the study results (0–25 %). Even when sensitivity analyses were undertaken, these seldom included varying the assumptions made about the underlying missing data mechanism. Examples of this included cases where the primary analyses utilised a complete case analysis and the associated sensitivity analyses consisted of single/multiple imputation or repeated measures models, or vice versa, or the addition of all variables that had been identified to be predictive of missing data into the analysis model.

Very few examples utilising the reasons for missing data in the imputation of missing values were identified, including the substitution of missing values in the EQ-5D-3L index for those who had died with zeros (i.e. the EQ-5D-3L health state equal to being dead) [41], using QLQ-C30 averages for missing data due to administration errors and lower scores for missing data due to refusal, illness, death [42] and imputing missing data with the best and worst observed scores [43] in order to assess the effect of a MNAR assumption on their results. However, none of these single imputation techniques took into account the uncertainty around the imputed values.

### Subset of articles using PROMs as a primary endpoint

The above summaries considered publications utilising the relevant PROMs as either a primary or secondary outcome. When focussing on the subset of articles utilising the relevant PROMs as a primary outcome measure only (80 PROMs, approximately one-third of all PROMs and 24–69 % of each relevant PROMs category), the standard of reporting improved marginally. More specifically, for some of the PROMs, an increase in the proportion of studies mentioning methods for reducing the amount of missing data within the studies could be observed, along with an increase in the clarification of how much PROMs data are available at the primary follow-up point and an overall decrease of the amount of missing data at follow-up. Overall, the proportion of articles that performed and reported sensitivity analyses increased. On the other hand, the proportion of studies using LOCF in their primary analysis and not clearly stating their analysis population also increased when only considering studies using relevant PROMs as a primary outcome measure.

## Discussion

This research shows that despite the wide availability of published guidance on this topic, the handling, analysis and reporting of missing PROM data in RCTs often failed to follow the current recommended best practice. Many authors did not comply with basic advice about the reporting of missing outcome data in RCTs, as also found in the previous reviews [15–22]. A lack of adequate reporting on attrition, i.e. missing data due to loss to follow-up in RCTs, was also discussed by Hopewell et al. [44].

Particularly noticeable in the present survey was the failure of many publications to describe clearly the extent of missing PROMs outcome data. CONSORT diagrams detailing the number of participants who died or were lost to follow-up did not capture the amount of missing data that occur due to questionnaire non-compliance or partly/incorrectly completed questionnaires. This, together with the lack of clarity on how missing data were handled in the analysis, made it impossible for the reader to assess the risk of bias arising from missing data in the reported results. Where missing data occurred partly by design (i.e. only a subgroup of participants was included into the PROMs research, because participants with disease progression or other patient characteristics are excluded, or because of a high mortality rate in the study making the collection of PROMs impossible for a large proportion of participants [45]), authors ought to ensure that results and interpretations are provided within this context, instead of extrapolating the conclusions inappropriately to the entire trial population.

In addition, the continued use of imputation methods that are known to introduce bias, such as LOCF [46, 47], further puts into question the validity of some study results.

Furthermore, there is limited evidence of repeatedly measured outcome data being taken into account for the PROMs analysis when it may be very informative for the imputation process.

The importance of sensitivity analysis to assess the robustness of the study results with regard to the untestable assumptions about the underlying missing data mechanism has been highlighted repeatedly in the literature [6, 7, 10, 48, 49]. The results presented here showed that sensitivity analysis has only been described in a low percentage of articles. Even where sensitivity analysis has been performed, the sensitivity of the assumptions made about missing data in the primary analysis was often not investigated, as suggested in the current literature [10], thus making it impossible for the reader to assess the robustness of results in relation to variations about the assumed missing data mechanism. As there was evidence of different rates of loss to follow-up by trial arm in many trials, there may be a need to consider MNAR mechanisms.

The potential influence of missing data on study results was rarely discussed, thus leaving the study results open to misinterpretation.

Finally, the number of publications reporting the methods to minimise the occurrence of missing data used in planning and conducting the study was found to be low. This is disappointing since no statistical analysis, however advanced, can replace information obtained by more complete follow-up. Therefore, researchers should be aware that in dealing with missing data ‘the single best approach is to prospectively prevent missing data occurrence’ [10].

## Strength and limitations of the study

This review adds to the current literature by focusing on recent publications and offering additional, very important aspects to the assessment of the handling and reporting of missing data in RCTs. Novel aspects included an investigation into the reporting of steps taken to minimise the occurrence of missing data and whether differential missing data rates by trial arm were considered in the analysis and reporting of the trial, as well as a justification of the chosen method for dealing with missing data and the use of sensitivity analysis.

By attempting to create a broad picture of current practice through including publications from a wide range of journals, rather than focussing on specific journals only, as in some of the previous reviews [15, 16, 20, 21], it was necessary to limit the review to a certain number of outcome measures. Though it is hoped that the reporting practice observed in the subset of representative outcome measures is generalisable to other PROMs, it is possible that there may be PROMs for which the handling, analysis and reporting of missing data is different from the standard of reporting as presented here.

Only very few eligible studies were identified for some PROMs (especially, the OHS and OKS, with four and nine studies, respectively, included in the review). Reasons for this included the fact that these site-specific measurements are just two of many other PROMs designed to be used for similar assessments [50–52]. Additionally, the pool of studies utilising these PROMs will naturally be smaller than for PROMs designed to measure a broader range of disease areas. Arguably, the low numbers of articles identified produced a less generalisable picture of the analysis and reporting practice of RCTs utilising these PROMs.

Generalisability is also limited to larger RCTs (due to the inclusion criteria of ≥50 participants per arm) and may not apply to the large amount of RCTs conducted that do not meet this sample size, including many single-centre studies, which are likely to differ from larger multicentre studies in terms of data collection, attrition and analysis methods.

The NHS EED database was included into the search strategy for the EQ-5D-3L and HUI, as it was considered to be very reliable in identifying the utility questionnaires. However, NHS EED relies on articles having been reviewed by the York team, and therefore, the entries for 2013 may not have been as up to date at the time of the review as the entries for earlier years would have been.

The follow-up periods in this review ranged from a few months to several years, as shown in Table 2. This may have been one of the reasons for the large variety in the observed extent of loss to follow-up.

The focus of this review was on the handling and reporting of missing PROMs outcome data, and missing data at baseline have not been within the remit of this research. Although less prevalent in RCTs than in epidemiological studies, it is recognised that missing baseline data also have the potential of biasing a study and certainly reduce the power in a complete case analysis. Therefore, authors should carefully consider how to report missing baseline data in their analyses, and multiple imputation approaches in line with the current literature may be advisable.

How authors reported potentially conflicting results from the primary and sensitivity analyses was not assessed because the review did not include sufficient numbers of appropriate sensitivity analyses to extract any meaningful information.

This work has not been able to relate the quality of reporting to word limits imposed by journals which may contribute to important details about missing data being omitted in favour of other relevant information. However, much of the information on data availability and analysis populations can be depicted in the tables and well-designed CONSORT flow charts. Details of assumptions about missing data mechanisms, analysis strategy and sensitivity analysis can be reported briefly with one or two sentences in the main text.

## Conclusions

This review provides evidence that a considerate discrepancy exists between the guidance and methodology on the handling, analysis and reporting of studies with missing PROMs outcome data compared to current practice in the publications of RCTs. The substandard level of reporting makes it challenging for clinicians, healthcare providers and policy makers to know how reliable the results from RCTs are, and may even lead to healthcare decisions being based on sub-optimal information.

Greater awareness needs to be created about the potential bias introduced by the inappropriate handling of missing data and the importance of sensitivity analysis. Subsequently, the handling of missing data, especially in PROMs, as well as its detailed and consistent reporting needs to be improved to adhere with current methodology and hence enable an appropriate assessment of any treatment effects and the associated conclusions in the publications of RCTs. Ensuring that researchers trained in statistics are among the authors and involved in the study design is thought to contribute to improving standards.

## Electronic supplementary material

Below is the link to the electronic supplementary material.

Supplementary material 1 (DOCX 11 kb)

## Abbreviations

EQ-5D-3L: EuroQol 5 Dimension 3-Level Questionnaire
HUI: Health Utility Index
NHS EED: NHS Economic Evaluation Database
OHS: Oxford Hip Score
OKS: Oxford Knee Score
PDQ: Parkinson’s Disease Questionnaire
PROMs: Patient-reported outcome measures
PROs: Patient-reported outcomes
QLQ-C30: European Organization for Research and Treatment of Cancer Quality of Life Questionnaire-Core 30
RCT: Randomised controlled trial
