# Supplementary material for: The current practice of handling and reporting missing outcome data in eight widely used PROMs in RCT publications: a review of the current literature
Source: Qual Life Res. 2016 Jan 28;25:1613–23. doi: 10.1007/s11136-015-1206-1 (PMC4893363; doi:10.1007/s11136-015-1206-1)
Supplement: Supplementary file 1 — Supplementary material 1 (DOCX 11 kb) [file 11136_2015_1206_MOESM1_ESM.docx]

**Information that was extracted from each eligible research article:**

year of publication • PROM used as a primary or secondary outcome • methods used to limit the amount of missing data • missing data mentioned in methods/ analysis section • assessment of differential rates of missing data in terms of baseline characteristics • number of participants randomised to the two relevant trial arms • proportion of available data at the primary assessment time point (defined as number of participants with available data divided by all participants randomised at either the final follow-up time point of the trial, or the time point specifically declared to be of main interest) • type of follow-up (repeated assessments vs. single follow-up) • length of follow-up until the primary assessment time point • analysis population • primary method of handling missing data• description of the assumed missing data mechanism • justification provided for primary method of dealing with missing data • performance of sensitivity analysis • discussion of the potential influence of missing data on the study results
